# Author Correction: The 4D Nucleome Data Portal as a resource for searching and visualizing curated nucleomics data

**DOI:** 10.1038/s41467-022-34458-4

**Published:** 2022-11-02

**Authors:** Sarah B. Reiff, Andrew J. Schroeder, Koray Kırlı, Andrea Cosolo, Clara Bakker, Luisa Mercado, Soohyun Lee, Alexander D. Veit, Alexander K. Balashov, Carl Vitzthum, William Ronchetti, Kent M. Pitman, Jeremy Johnson, Shannon R. Ehmsen, Peter Kerpedjiev, Nezar Abdennur, Maxim Imakaev, Serkan Utku Öztürk, Uğur Çamoğlu, Leonid A. Mirny, Nils Gehlenborg, Burak H. Alver, Peter J. Park

**Affiliations:** 1 Department of Biomedical Informatics, Harvard Medical School, Boston, MA 02115 USA; 2 Institute for Medical Engineering and Science, Massachusetts Institute of Technology, Cambridge, MA 02139 USA; 3 Karya SMD Software Solutions, İzmir, 35040 Turkey; 4 Department of Physics, Massachusetts Institute of Technology, Cambridge, MA 02139 USA

**Keywords:** Genetic databases, Computational platforms and environments, Genomics

Correction to: *Nature Communications* 10.1038/s41467-022-29697-4, published online 02 May 2022

The original version of this Article omitted from the author list the 6th author, Luisa Mercado, who is from the Department of Biomedical Informatics, Harvard Medical School. This has been corrected in both the PDF and HTML versions of the Article. This author was already included in the Author Contributions as L.M. and therefore no further corrections to this statement were made.

