# Supplementary material for: The 4D Nucleome Data Portal as a resource for searching and visualizing curated nucleomics data
Source: Nat Commun. 2022 May 2;13:2365. doi: 10.1038/s41467-022-29697-4 (PMC9061818; doi:10.1038/s41467-022-29697-4)
Supplement: Supplementary file 2 — Reporting Summary [file 41467_2022_29697_MOESM2_ESM.pdf]

nature portfolio

Corresponding author(s): Peter J. Park

Last updated by author(s): Mar 3, 2022

Reporting Summary

Nature Portfolio wishes to improve the reproducibility of the work that we publish. This form provides structure for consistency and transparency in reporting. For further information on Nature Portfolio policies, see our Editorial Policies and the Editorial Policy Checklist.

Statistics

For all statistical analyses, confirm that the following items are present in the figure legend, table legend, main text, or Methods section.

n/a	Confirmed	
☒	☐	The exact sample size (*n*) for each experimental group/condition, given as a discrete number and unit of measurement
☒	☐	A statement on whether measurements were taken from distinct samples or whether the same sample was measured repeatedly
☒	☐	The statistical test(s) used AND whether they are one- or two-sided<br>*Only common tests should be described solely by name; describe more complex techniques in the Methods section.*
☒	☐	A description of all covariates tested
☒	☐	A description of any assumptions or corrections, such as tests of normality and adjustment for multiple comparisons
☒	☐	A full description of the statistical parameters including central tendency (e.g. means) or other basic estimates (e.g. regression coefficient) AND variation (e.g. standard deviation) or associated estimates of uncertainty (e.g. confidence intervals)
☒	☐	For null hypothesis testing, the test statistic (e.g. *F*, *t*, *r*) with confidence intervals, effect sizes, degrees of freedom and *P* value noted<br>*Give P values as exact values whenever suitable.*
☒	☐	For Bayesian analysis, information on the choice of priors and Markov chain Monte Carlo settings
☒	☐	For hierarchical and complex designs, identification of the appropriate level for tests and full reporting of outcomes
☒	☐	Estimates of effect sizes (e.g. Cohen's *d*, Pearson's *r*), indicating how they were calculated

Our web collection on statistics for biologists contains articles on many of the points above.

Software and code

Policy information about availability of computer code

Data collection	Data submission was performed using our python command line tool Submit4DN (https://github.com/4dn-dcic/Submit4DN, current version 2.0.3). Further information about usage of Submit4DN is available at https://data.4dnucleome.org/help/submitter-guide.
Data analysis	The front-end code is https://github.com/4dn-dcic/fourfront (current version 3.6.1). The database code is https://github.com/4dn-dcic/snovault (current version 5.2.0). All of our automated analysis pipelines are available on github. The Hi-C pipeline is available at https://github.com/4dn-dcic/docker-4dn-hic (v43); the Repliseq pipeline is available at https://github.com/4dn-dcic/docker-4dn-repliseq (v16); the CUT&RUN pipeline is available at https://github.com/4dn-dcic/docker-4dn-cut-and-run-pipeline/tree/v1 (v1); the MARGI pipeline is available at https://github.com/4dn-dcic/iMARGI-Docker (v1.1.1); the RNA-seq pipeline is available at https://github.com/4dn-dcic/rna-seq-pipeline (v1.1); the ATAC-seq pipeline is available at https://github.com/4dn-dcic/atac-seq-pipeline (v1.1.1); the ChIP-seq pipeline is available at https://github.com/4dn-dcic/chip-seq-pipeline2 (v1.1.1).The code for calling insulation scores and boundaries is at https://github.com/4dn-dcic/docker-4dn-insulation-scores-and-boundaries-caller/tree/v1 (v1). For QC we used FastQC v0.11.9 and PairsQC v0.2.6. Descriptions of each pipeline are available at https://data.4dnucleome.org/resources/data-analysis. For managing and running pipelines we use Tibanna FF (https://github.com/4dn-dcic/tibanna_ff/), v0.23.2.

For manuscripts utilizing custom algorithms or software that are central to the research but not yet described in published literature, software must be made available to editors and reviewers. We strongly encourage code deposition in a community repository (e.g. GitHub). See the Nature Portfolio guidelines for submitting code & software for further information.

Data

Policy information about availability of data

All manuscripts must include a data availability statement. This statement should provide the following information, where applicable:

- Accession codes, unique identifiers, or web links for publicly available datasets
- A description of any restrictions on data availability
- For clinical datasets or third party data, please ensure that the statement adheres to our policy

All datasets described are publicly available at https://data.4dnucleome.org/. All datasets present in the 4D Nucleome data portal were submitted either by the researchers who generated the data or by 4DN curators transferring some datasets from GEO. Analysis was performed using the genome references GRCh38 for humans (https://www.encodeproject.org/files/GRCh38_no_alt_analysis_set_GCA_000001405.15/) and GRCm38 for mouse (https://www.encodeproject.org/files/mm10_no_alt_analysis_set_ENCODE/@@download/mm10_no_alt_analysis_set_ENCODE.fasta.gz).

Field-specific reporting

Please select the one below that is the best fit for your research. If you are not sure, read the appropriate sections before making your selection.

[x] Life sciences [ ] Behavioural & social sciences [ ] Ecological, evolutionary & environmental sciences

For a reference copy of the document with all sections, see nature.com/documents/nr-reporting-summary-flat.pdf

Life sciences study design

All studies must disclose on these points even when the disclosure is negative.

Sample size	Experiments not performed; this manuscript describes a data repository.
Data exclusions	Experiments not performed; this manuscript describes a data repository.
Replication	Experiments not performed; this manuscript describes a data repository. However, we encourage submitters to perform experiments with biological replicates and make this information available in our repository.
Randomization	Experiments not performed; this manuscript describes a data repository.
Blinding	Experiments not performed; this manuscript describes a data repository.

Reporting for specific materials, systems and methods

We require information from authors about some types of materials, experimental systems and methods used in many studies. Here, indicate whether each material, system or method listed is relevant to your study. If you are not sure if a list item applies to your research, read the appropriate section before selecting a response.

Materials & experimental systems

n/a	Involved in the study	
[x]	[ ]	Antibodies
[x]	[ ]	Eukaryotic cell lines
[x]	[ ]	Palaeontology and archaeology
[x]	[ ]	Animals and other organisms
[x]	[ ]	Human research participants
[x]	[ ]	Clinical data
[x]	[ ]	Dual use research of concern

Methods

n/a	Involved in the study	
[x]	[ ]	ChIP-seq
[x]	[ ]	Flow cytometry
[x]	[ ]	MRI-based neuroimaging
